# A non-functional 5′ *ALK* fusion validated at the RNA level as a classical *EML4-ALK* that responds well to the novel *ALK* inhibitor ensartinib: A case report

**DOI:** 10.3389/fmed.2022.979032

**Published:** 2022-10-06

**Authors:** Hong Yang, Haojing Li, Yu Fang, Zhijun Li, Jianhua Zhu, Huan Liu, Chao Lu, Xiaoyan Zhang, Tonghui Ma, Cuiying Zhang

**Affiliations:** ^1^Department of Oncology, Inner Mongolia People’s Hospital, Hohhot, China; ^2^Department of Translational Medicine, Genetron Health (Beijing) Technology, Co., Ltd., Beijing, China; ^3^Department of Oncology, Inner Mongolia Medical University, Hohhot, China

**Keywords:** 5′ NOK fusion, *ALK*, RNA NGS, ensartinib, NSCLC

## Abstract

**Background:**

Currently, many targeted drugs are approved for treatment of *ALK* fusion non-small cell lung cancer. However, it has been previously assumed that patients with 5′ non-oncogenic kinase (5′ NOK) fusion detected by DNA next-generation sequencing (NGS) would not benefit from *ALK* inhibitors because of lack of an intact kinase domain.

**Case description:**

A novel 5′ NOK fusion form, *ALK-CYP27C1* (A19:C5), was detected by DNA NGS in surgical tissue specimens of a patient with recurrent lung adenosquamous carcinoma. The patient achieved 29 months of progression-free survival with ensartinib treatment. The results of RNA NGS from the same operative tissue identified *EML4-ALK* (E13:A20) fusion variant type I.

**Conclusion:**

This is the first case to provide real-world evidence of effective treatment of a patient with the 5′ NOK fusion form at the DNA level but functional *EML4-ALK* at the RNA level, illustrating the need for RNA testing in 5′ NOK patients.

## Introduction

Lung cancer is one of the most common cancer types in China and is the leading cause of cancer death (1). With the development of targeted drugs, an increasing number of patients whose tumors harbor driver mutations, especially fusions, can receive targeted therapy. *ALK* fusion is an important driver mutation that accounts for approximately 3-7% of non-small cell lung cancer (NSCLC) cases (2–4), and the most common form is *EML4-ALK* (5, 6). Conventional diagnostic strategies for fusion include immunohistochemistry (IHC) and fluorescence *in situ* hybridization (FISH); however, both are low-throughput (7). Additionally, a previous report has shown that the false-negative rate of FISH and IHC is 24 and 12.6%, respectively, which may lead to patients not receiving ALK inhibitor treatment in time (8). A total of 303 patients with ALK IHC-positive NSCLC were reevaluated in the ALEX trial study, of which 203 were FISH-positive, 39 were FISH-negative, and 61 were FISH-uninformative, and the corresponding ORR was 90.6, 28.6, and 96%, respectively (9). This suggested that the results were differences between DNA level (FISH) and protein level (IHC) in fusion detection, and that the patients with ALK IHC-positive and FISH-negative or ALK IHC-positive and FISH-uninformative results could also benefit from ALK inhibitors.

NGS has been recommended by the National Comprehensive Cancer Network (NCCN) guidelines for genetic testing. Because of the high throughput of DNA NGS detection techniques, driver mutations are more easily detected with these approaches than by FISH or IHC. Meanwhile, more fusion forms of the *ALK* gene have been identified, including novel partners and breakpoints, some of which may be transcribed to non-functional or other fusion forms at the RNA level (10–12). 5′ NOK fusion is a novel form at the DNA level that does not have an intact kinase domain; therefore, it has been suggested that patients with such a fusion cannot benefit from ALK inhibitor treatment.

Here, we report a case of lung adenosquamous carcinoma that recurred after surgical resection, harboring a novel 5′ NOK fusion form, *ALK-CYP27C1*, which benefited from the *ALK* inhibitor ensartinib. RNA NGS revealed that it was *EML4-ALK* (E13:A20, V1), which suggested that further detection is required at the RNA level when a single 5′ NOK is revealed by DNA NGS.

## Case description

**FIGURE 1:**
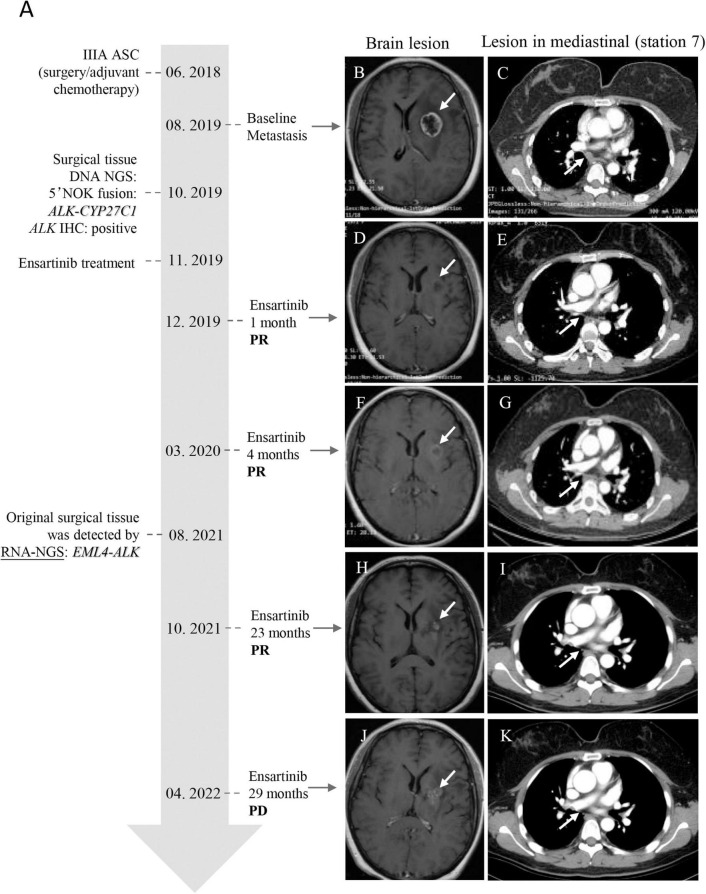
Timeline of the 5′ NOK *ALK* fusion clinical diagnosis and treatment and MRI/X-ray computed tomography of lesions of the brain and mediastinal lymph node (station 7) of the patient. **(A)** Timeline of patient clinical diagnosis and treatment. **(B,C)** Baseline: brain lesion (24*21 mm) and lymphadenopathy in the mediastinum (station 7) (short diameter, 10 mm). **(D,E)** One month after ensartinib treatment: brain lesion reduction (18*14 mm) and the lesion in the mediastinum (station 7) was reduced (short diameter, 4 mm). **(F,G)** Four months after ensartinib treatment: brain lesion reduction (16*15 mm) and the lesion in the mediastinum (station 7) disappeared. **(H,I)** Twenty-three months after ensartinib treatment: the brain lesion shrank again (15.8*13.8 mm), and there was no lesion in the mediastinal region (station 7). **(J,K)** Twenty-nine months after ensartinib treatment: the brain lesion progressed (28*21 mm), but the mediastinal region (station 7) was still responding. The white arrow indicates the lesion. ASC: adenosquamous carcinoma; PR: partial response; PD: progressive disease.

**FIGURE 2:**
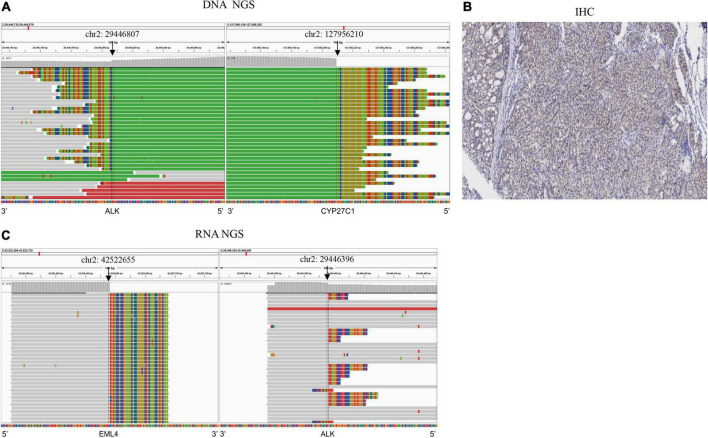
Multiple methods were used to detect *ALK* fusions in the surgical tissue. **(A)** *ALK-CYP27C1* fusion was detected by DN ANGS; **(B)** *ALK* protein expression was detected by IHC. **(C)** *EML4-ALK* fusion was detected by RNA NGS.

The patient was a 49-year-old Mongolian female with no history of smoking. She was admitted to our hospital with a cough of 2 months. After systematic examination and immunohistochemistry, adenocarcinoma of the right lung was considered. She then underwent a right middle and lower lobectomy in June 2018. The postoperative pathology indicated lung adenosquamous carcinoma, and the pathologic stage was IIIA; thus, she accepted pemetrexed plus carboplatin as standard adjuvant chemotherapy (Figure 1A). However, 14 months after the operation, in August 2019, she was referred to the hospital for headache. The brain MRI revealed a nodule in the left basal ganglia with edema, compression of the ventricle, and right midline shift (Figure 1B). The chest CT showed lymphadenopathy in the mediastinum (station 7) (Figure 1C). The patient then received radiotherapy for the brain lesion in September 2019. Mannitol and a small dose of dexamethasone were administered to reduce intracranial pressure, and the headache symptoms were slightly relieved, but the brain lesion did not shrink significantly (data not shown). To determine the next treatment, in October 2019, DNA NGS was performed on her previous surgical tissue specimen using OncoPanscan™ at the Diagnostic Laboratory of Genetron Health (Supplementary Data 1). A 5′ NOK fusion *ALK-CYP27C1* (A19:C5) was identified in DNA level, of which a breakpoint occurred in chromosome 2: 29,446,807 (intron 19-*ALK*) and chromosome 2: 127,956,210 (intron 4-*CYP27C1*) (Figure 2A). The *ALK* fusion part was in the 5-terminal region without a kinase domain, which was previously considered to not express the *ALK* protein. The *ALK* fusion was validated by another DNA panel, Oncofocus™ (Genetron Health) (Supplementary Figure 1 and Supplementary Data 1). To confirm whether the *ALK* protein was expressed, IHC was further performed on a Ventana platform, and *ALK* protein expression was positive (Figure 2B). According to the positive results of ALK IHC, the patient was enrolled in a donation project about a new *ALK* inhibitor, ensartinib, on 19 November 2019, and received 225 mg orally once daily. One week after the treatment, the dizziness and headache symptoms were relieved, and no adverse events were observed. The brain and mediastinal lesions (station 7) were significantly reduced after 1 month of medication (Figures 1D,E; December 2019). The chest lymph node disappeared after an additional 3 months of ensartinib treatment (Figures 1F,G; March 2020). The brain lesion shrank again after 23 months of ensartinib treatment (Figures 1H,I; October 2021). Because different ALK fusions affect the efficacy of ALK inhibitors differently, previous articles have reported differences between DNA NGS and RNA NGS in fusion detection, and RNA NGS had become increasingly popular in recent years. We hoped to perform RNA NGS detection to guide subsequent treatment. In addition, we also wanted to explore the cause of efficient treatment of ensartinib against the 5′ NOK fusion of *ALK*. Therefore, RNA NGS was conducted on the same surgical tissue specimen using Fusioncapture™ (Genetron Health) in August 2021, which involved all exons of the *ALK* gene for any type of *ALK* fusion detected (Supplementary Data 2). Intriguingly, the result showed *EML4-ALK* (V1, E13:A20) instead of *ALK-CYP27C1* (Figure 2C). When the patient had been using ensartinib for 29 months, the brain lesion began to progress, while mediastinal lesions (station 7) continued to respond (Figures 1J,K, April 2022). Following 29 months of clinical and radiological response, the patient experienced disease progression in the brain only; following stereotactic radiotherapy, the patient resumed therapy but had clinical progression. The patient did not feel unwell during follow-up.

## Discussion

Here, we present a case of postoperative recurrent lung adenosquamous carcinoma in which a surgical tissue was identified by DNA NGS as a novel form of 5′ NOK fusion, *ALK-CYP27C1* (A19:C5) while also being positive for the *ALK* protein by IHC. Treatment with the *ALK* inhibitor ensartinib was administered for nearly 29 months until the brain lesion progressed. Then, the patient received local stereotactic radiotherapy and continued to take ensartinib. The effect was similar to the results of a randomized clinical trial of ensartinib on patients with *ALK*-positive NSCLC that showed a median PFS of 25.8 months (13).

To the best of our knowledge, this is the first report on a 5′NOK form of *ALK* fusion in DNA NGS that benefited from an *ALK* inhibitor (ensartinib). Subsequently, the same surgical tissue specimen was further verified by RNA NGS. No *ALK-CYP27C1* fusion was detected at the RNA level, but *EML4-ALK* (V1, E13:A20), a common fusion form of *ALK*, was detected. Recent studies have shown that clinical benefits were different among various forms of *EML4-ALK*, and that *EML4-ALK* (E13:A20, V1) can respond well to *ALK* inhibitors, including ensartinib (5, 14, 15). In the present case, the patient was diagnosed as having an *EML4-ALK* (E13:A20, V1) fusion at the RNA level. This may be the reason the patient responded well to ensartinib. Here, we found for the first time a 5′ NOK fusion that could have resulted in a functional *EML4-ALK* transcript and responded well to the *ALK* inhibitor ensartinib.

Although various techniques can be used to identify gene fusion, including IHC, FISH, DNA NGS, and RNA NGS, each technique has its own limitations. The ALEX trial showed that because of low sensitivity, the result of DNA level detection by FISH was not enough for fusion detection and required a verification by protein level detection (i.e., IHC) (9), and that IHC was not as specific as FISH (3, 7). Moreover, the detection flux of FISH and IHC is very low. The NGS technology has the advantage of high-throughput, and DNA NGS has been widely conducted in clinical practice. However, previous reports showed that DNA NGS results were different from RNA NGS results in fusion detection, such as many uncommon fusion forms (intergenic-breakpoint fusions, novel partner fusions, and intragenic fusions) identified at the DNA level by DNA NGS, had been verified as common fusions, or uncommon functional fusions, or no transcription at the RNA level (16, 17). For RNA NGS, high-quality RNA is also difficult to obtain (3). The reasons for different fusion being detected at the DNA and RNA levels in the same sample are unclear. A possible explanation may be chromothripsis, which can result in thousands of chromosomal rearrangements (18). In this case, *ALK-CYP27C1* has been detected by DNA NGS, while *EML4-ALK* was found at the RNA level, which may due to *EML4-ALK* rearrangement being mediated by chromothripsis. Based on the evidence of previous reports and this case, we suggest that validation of the RNA level is needed for fusions without an intact kinase domain. These results indicate that a combination of multiple methods, especially DNA NGS and RNA NGS, is necessary for fusion detection.

## Conclusion

To our knowledge, this is the first case of a 5′ NOK fusion that benefited from an *ALK* inhibitor. This demonstrates the necessity of further analysis from the RNA level of 5′ NOK fusion per DNA NGS to identify whether there is another functional structural form. Precise analysis of such a fusion (5′ NOK) form may yield clinical benefits for patients. In clinical practice, it would be better to analyze DNA and RNA simultaneously to reduce the turnaround time.

## Data availability statement

The original contributions presented in this study are included in the article/Supplementary material, further inquiries can be directed to the corresponding author/s.

## Author contributions

CZ, TM, and XZ contributed to conception and design of the study. HY and YF wrote the first draft of the manuscript. HAL and ZL prepared figures and background research. JZ prepared figures. HUL and CL prepared background research. All authors contributed to manuscript revision, read, and approved the submitted version.

## Supplementary material

Supplementary Figure 1 *ALK-CYP27C1* fusion was validated by DNA NGS in another DNA panel.
