# Supplementary material for: A non-functional 5′ ALK fusion validated at the RNA level as a classical EML4-ALK that responds well to the novel ALK inhibitor ensartinib: A case report
Source: Front Med (Lausanne). 2022 Oct 6;9:979032. doi: 10.3389/fmed.2022.979032 (PMC9582288; doi:10.3389/fmed.2022.979032)
Supplement: Supplementary file 1 [file Table_1.DOCX]

**Supplemental data 1: The** **technical specifics of DNA NGS**

Genomic DNA was isolated from tumor samples using QIAamp DNA FFPE tissue kit (Qiagen), and the concentrations determined using a Qubit 3.0 Fluorometer (Thermo Fisher Scientific). Then the genomic DNA was fragmented for constructing a library using KAPA Hyper Prep kits (KAPA, KK8504). The DNA libraries were analyzed using OncoPanscan^TM^ and Oncofocus^TM^ (Genetron Health).

| **The gene list of the OncoPanscan^TM^ panel** | | | | | | | |
| --- | --- | --- | --- | --- | --- | --- | --- |
| ALK | ABCB1 | ABCC4 | ABL1 | AKT1 | AKT3 | APC | ARAF |
| ARID1A | ATM | BMPR1A | BRAF | BRCA1 | BRCA2 | BRIP1 | BTK |
| C8orf34 | CBR3 | CDA | CDH1 | CDK4 | CDK6 | CDKN2A | CHEK1 |
| CHEK2 | CSF1R | CTNNB1 | CYP2B6 | DHFR | DNMT3A | DPYD | DYNC2H1 |
| EGFR | EPCAM | EPHA5 | ERBB2 | ERBB3 | ERBB4 | ERCC1 | ERCC2 |
| ESR1 | ETV6 | FBXW7 | FGFR1 | FGFR2 | FGFR3 | FGFR4 | FLT3 |
| FLT4 | GNA11 | GNAQ | GNAS | GGH | GSTP1 | HRAS | IDH1 |
| IDH2 | IGF1R | JAK1 | JAK2 | KDR | KIT | KRAS | MAP2K1 |
| MDM2 | MET | MLH1 | MLH3 | MSH2 | MSH6 | MTHFR | MTR |
| MUTYH | MYC | NAT2 | NF1 | NF2 | NRAS | NRG1 | NTRK1 |
| NTRK2 | NTRK3 | PALB2 | PDGFRA | PDGFRB | PIK3CA | PIK3R1 | PMS1 |
| PMS2 | POLD1 | POLE | PTCH1 | PTCH2 | PTEN | RAF1 | RB1 |
| RET | ROS1 | SDHA | SDHB | SDHC | SDHD | SLC22A16 | SMAD4 |
| SMO | SOD2 | SRC | STK11 | TERT | TP53 | UGT1A1 | UMPS |
| VHL | VEGFA | XPC | XRCC1 |  |  |  |  |

| **The gene list of the Oncofocus^TM^ panel** | | | | | | | |
| --- | --- | --- | --- | --- | --- | --- | --- |
| ABL1 | AKT1 | ALK | APC | AR | ATM | BCR | BRAF |
| CDH1 | CDK4 | CDK6 | CDKN2A | CSF1R | CTNNB1 | DNMT3A | EGFR |
| ERBB2 | ERBB4 | ESR1 | EZH2 | FBXW7 | FGFR1 | FGFR2 | FGFR3 |
| FLT3 | GNA11 | GNAQ | GNAS | HNF1A | HRAS | IDH1 | IDH2 |
| JAK2 | JAK3 | KDR | KIT | KRAS | MAP2K1 | MET | MLH1 |
| MPL | MYC | NPM1 | NTRK1 | NRAS | PDGFRA | PDGFRB | PIK3CA |
| PIK3R1 | PTEN | PTPN11 | RARA | RB1 | RET | ROS1 | SMAD4 |
| SMARCB1 | SMO | SRC | STK11 | TERT | TP53 | VHL |  |
