# Supplementary material for: A non-functional 5′ ALK fusion validated at the RNA level as a classical EML4-ALK that responds well to the novel ALK inhibitor ensartinib: A case report
Source: Front Med (Lausanne). 2022 Oct 6;9:979032. doi: 10.3389/fmed.2022.979032 (PMC9582288; doi:10.3389/fmed.2022.979032)
Supplement: Supplementary file 2 [file Table_2.DOCX]

**Supplemental data 2: The** **technical specifics of RNA NGS**

Total RNA was extracted using the AllPrep DNA/RNA Mini Kit (Qiagen), and reverse transcribed using SuperScript III Reverse Transcriptase (Thermo Fisher Scientific). The DNA libraries were constructed with the KAPA HTP Library Preparation Kit (KAPA Biosystems) and analyzed using Fusioncapture (Genetron Health).

| **The gene list of the Fusioncapture^TM^ panel** | | | | | | | |
| --- | --- | --- | --- | --- | --- | --- | --- |
| ABI1 | ABL1 | ABL2 | ACSL6 | ADCY9 | AFF1 | AFF3 | AFF4 |
| AKT3 | ALK | ARHGAP26 | ARHGEF12 | ARID1A | ARNT | ASXL1 | ASXL2 |
| ATF1 | ATF7IP | ATG5 | ATIC | AUTS2 | AXL | BAALC | BCAT1 |
| BCL10 | BCL11A | BCL11B | BCL2 | BCL3 | BCL6 | BCL7A | BCL9 |
| BCOR | BCR | BIRC3 | BLNK | BMF | BRAF | BTG1 | BTK |
| C15ORF65 | C1orf43 | CAMTA1 | CARS | CBFA2T3 | CBFB | CBL | CCNB3 |
| CCND1 | CCND2 | CCND3 | CD22 | CD274 | CD28 | CD79B | CDK6 |
| CDKN2A | CDX2 | CEBPA | CEBPE | CEP85L | CHD1 | CHIC2 | CHMP2A |
| CHN1 | CHST11 | CIC | CIITA | CLP1 | CLTC | CLTCL1 | CNTRL |
| COL1A1 | CREB1 | CREB3L1 | CREB3L2 | CREBBP | CRLF2 | CSF1 | CSF1R |
| CSF3R | CTCF | CTLA4 | CTNNB1 | CUX1 | DAZAP1 | DDIT3 | DDX10 |
| DDX6 | DEK | DENND3 | DGKH | DNMT3A | DUSP22 | DUX4 | EBF1 |
| EGFR | EIF2B1 | EIF4A2 | ELF4 | ELL | ELMO1 | ELN | EMC7 |
| EML4 | ENTPD1 | EP300 | EPOR | EPS15 | ERBB2 | ERG | ETS1 |
| ETV1 | ETV4 | ETV5 | ETV6 | EWSR1 | FBN3 | FBRSL1 | FBXW2 |
| FBXW7 | FCGR2B | FCRL4 | FER | FEV | FGFR1 | FGFR1OP | FGFR2 |
| FGFR3 | FIP1L1 | FLI1 | FLT3 | FLT3LG | FNBP1 | FOXO1 | FOXO3 |
| FOXO4 | FOXP1 | FSTL3 | FUS | FUT8 | GAS7 | GATA2 | GLI1 |
| GLIS2 | GMPS | GPHN | GPI | HAVCR2 | HERPUD1 | HEY1 | HIP1 |
| HIST1H4I | HLF | HMGA1 | HMGA2 | HNRNPUL1 | HOXA10 | HOXA11 | HOXA13 |
| HOXA3 | HOXA9 | HOXC11 | HOXC13 | HOXD11 | HOXD13 | HSP90AA1 | HSP90AB1 |
| IGHJ1 | IKZF1 | IKZF3 | IL16 | IL21R | IL2RB | IL3 | IQGAP2 |
| IRF4 | IRS4 | ITK | ITPKB | JAK1 | JAK2 | JAK3 | JAZF1 |
| KAT6A | KDM2B | KDM5A | KDM6A | KDSR | KIF5B | KLK11 | KMT2A |
| KMT2D | KRAS | LASP1 | LCK | LCP1 | LMO1 | LMO2 | LPP |
| LRMP | LYL1 | MAF | MAFB | MALT1 | MAP2K4 | MBNL1 | MDS2 |
| MECOM | MEF2D | MET | METRNL | MKL1 | MLF1 | MLLT1 | MLLT10 |
| MLLT11 | MLLT3 | MLLT4 | MLLT6 | MME | MN1 | MNX1 | MSI2 |
| MSN | MTCP1 | MUC1 | MYB | MYBL1 | MYC | MYH11 | MYH9 |
| NAB2 | NACA | NBEA | NCOA2 | NDRG1 | NEK6 | NF1 | NF2 |
| NFKB2 | NIN | NKX2-1 | NONO | NOTCH1 | NOTCH2 | NPM1 | NR4A3 |
| NRAS | NSD1 | NSD2 | NTRK1 | NTRK2 | NTRK3 | NUMA1 | NUP214 |
| NUP98 | NUTM2A | OLIG2 | OMD | P2RY8 | PAFAH1B2 | PAX3 | PAX5 |
| PAX7 | PBX1 | PCM1 | PCSK7 | PDCD1 | PDCD1LG2 | PDE4DIP | PDGFB |
| PDGFRA | PDGFRB | PER1 | PHF1 | PICALM | PIM1 | PIM3 | PLAG1 |
| PML | POU2AF1 | PPFIBP1 | PPP1CB | PRDM1 | PRDM16 | PRRC1 | PRRX1 |
| PSIP1 | PSMB2 | PSMB4 | PTCH1 | PTK2B | PTK7 | PTPN1 | PUM1 |
| PVT1 | RAB7A | RABEP1 | RAD51B | RAF1 | RALGDS | RANBP2 | RAP1GDS1 |
| RARA | RB1 | RBM15 | RCSD1 | REEP5 | RET | RHOH | RNF213 |
| ROS1 | RPL22 | RPN1 | RUNX1 | RUNX1T1 | RUNX2 | SEC31A | SEPT5 |
| SEPT6 | SEPT9 | SERPINA9 | SET | SETBP1 | SF3B1 | SH3BP5 | SH3GL1 |
| SKI | SLAMF7 | SLC1A2 | SNRPD3 | SNX29 | SPI1 | SQSTM1 | SRSF3 |
| SS18 | SS18L1 | SSBP2 | SSX1 | SSX2 | SSX4 | STAG2 | STAT5B |
| STAT6 | STIL | STRN3 | SYK | TAF15 | TAL1 | TAL2 | TBL1XR1 |
| TCF12 | TCF3 | TCF7 | TCL1A | TEC | TET1 | TFE3 | TFG |
| TFPT | TFRC | TLX1 | TLX3 | TMPRSS2 | TNFRSF11A | TNIP1 | TOP1 |
| TP53 | TP63 | TPM3 | TPM4 | TPR | TRIM24 | TRIM27 | TRIP11 |
| TTL | TYK2 | USP6 | VCP | VPS29 | WHSC1L1 | WT1 | WWTR1 |
| XIAP | YPEL5 | YWHAE | ZAP70 | ZBTB16 | ZCCHC7 | ZEB2 | ZFAND3 |
| ZMYM2 | ZNF384 | ZNF521 |  |  |  |  |  |
