# Supplementary figures and images for: A non-functional 5′ ALK fusion validated at the RNA level as a classical EML4-ALK that responds well to the novel ALK inhibitor ensartinib: A case report
Source: Front Med (Lausanne). 2022 Oct 6;9:979032. doi: 10.3389/fmed.2022.979032 (PMC9582288; doi:10.3389/fmed.2022.979032)

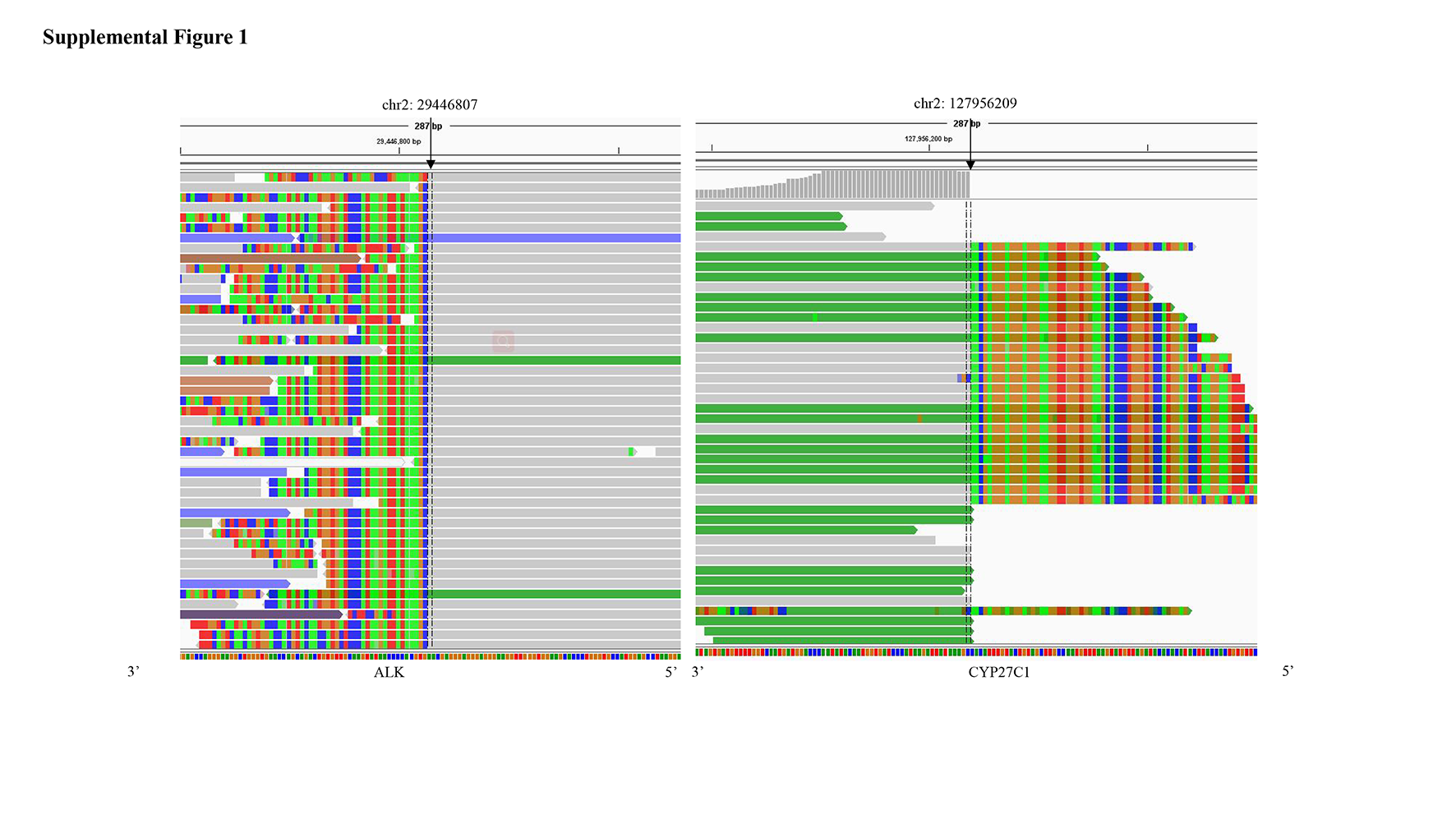

Supplement: Supplementary Figure 1 — ALK-CYP27C1 fusion was validated by DNA NGS in another DNA panel. [file Image_1.TIF]
